# High-Throughput Proteomic Approaches to the Elucidation of Potential Biomarkers of Chronic Allograft Injury (CAI)

**DOI:** 10.3390/proteomes1020159

**Published:** 2013-09-23

**Authors:** Hilary Cassidy, Jennifer Slyne, Helena Frain, Craig Slattery, Michael P. Ryan, Tara McMorrow

**Affiliations:** Renal Disease Research Group, School of Biomolecular and Biomedical Sciences, UCD Conway Institute, University College Dublin, Belfield, Dublin 4, Ireland; E-Mails: hilary.cassidy@ucdconnect.ie (H.C.); jennifer.slyne@ucdconnect.ie (J.S.); helena.frain@ucdconnect.ie (H.F.); craig.slattery@ucd.ie (C.S.); michael.p.ryan@ucd.ie (M.P.R.)

**Keywords:** chronic allograft injury, chronic allograft nephropathy, IF/TA, calcineurin inhibitors, cyclosporine, transplantation, proteomics, biomarkers

## Abstract

This review focuses on the role of OMICs technologies, concentrating in particular on proteomics, in biomarker discovery in chronic allograft injury (CAI). CAI is the second most prevalent cause of allograft dysfunction and loss in the first decade post-transplantation, after death with functioning graft (DWFG). The term CAI, sometimes referred to as chronic allograft nephropathy (CAN), describes the deterioration of renal allograft function and structure as a result of immunological processes (chronic antibody-mediated rejection), and other non-immunological factors such as calcineurin inhibitor (CNI) induced nephrotoxicity, hypertension and infection. Current methods for assessing allograft function are costly, insensitive and invasive; traditional kidney function measurements such as serum creatinine and glomerular filtration rate (GFR) display poor predictive abilities, while the current “gold-standard” involving histological diagnosis with a renal biopsy presents its own inherent risks to the overall health of the allograft. As early as two years post-transplantation, protocol biopsies have shown more than 50% of allograft recipients have mild CAN; ten years post-transplantation more than 50% of the allograft recipients have progressed to severe CAN which is associated with diminishing graft function. Thus, there is a growing medical requirement for minimally invasive biomarkers capable of identifying the early stages of the disease which would allow for timely intervention. Proteomics involves the study of the expression, localization, function and interaction of the proteome. Proteomic technologies may be powerful tools used to identify novel biomarkers which would predict CAI in susceptible individuals. In this paper we will review the use of proteomics in the elucidation of novel predictive biomarkers of CAI in clinical, animal and *in vitro* studies.

## 1. Introduction

The two most common causes of long-term graft loss remain “death with a functioning graft”, usually from a marked excess of cardiovascular mortality in allograft recipients, and chronic allograft injury (CAI), the term given to the development of fibrotic processes leading to progressive allograft dysfunction with variable proteinuria and hypertension [1,2]. It has been suggested that these two processes may represent the systemic and local manifestations of developing vascular disease (micro- and macro-) that are accelerated in the presence of a functioning transplant and transplant immunosuppression. Recent studies have demonstrated that progressive injury to the renal microvasculature is one of the first features of developing CAI [3] and this injury occurs almost uniformly by 10 years post transplantation and is associated with calcineurin-inhibitor (CNI) therapy [4]. It should also be noted that renal allograft failure is the fourth most common cause of End Stage Renal Disease (ESRD) in the United States [5,6]. In this review we focus on chronic allograft injury and cyclosporine nephrotoxicity and the ability of proteomic techniques to elucidate potential diagnostic biomarkers arising from these types of renal injuries.

## 2. Immunosuppressant—Induced Nephrotoxicity

Over the past 40 years, immunosupressive agents have revolutionised the world of solid organ transplantation. The use of these immunosuppressive agents prevents the progression of immune-mediated injury that occurs in transplanted organs. The general mechanisms of action of the currently employed immunosuppressive therapies operate by targeting T cell activation and cytokine activation [7]. Despite the obvious advantages associated with their use, it is well documented that both acute and chronic nephrotoxicity are associated complications of immunosuppressant regimes, particularly with the CNIs, namely cyclosporine A (CsA) and Tacrolimus (FK506) [8,9].

CsA exerts its immunosuppressive effect through the binding of the intracellular cytosolic immunophilin, cyclophilin, forming a complex which inhibits calcineurin phosphatase, thus preventing dephosphorylation of the NFAT family members responsible for the transcription of T-cell activating cytokines IL2 and IL4 [10,11]. Acute CsA nephrotoxicity has been documented within weeks of the initiation of CNI therapy, resulting in a decline in GFR coupled with decreased renal blood flow [9]. Acute CsA nephrotoxicity can generally be reversed simply by reducing or ceasing CsA therapy [12,13]. Chronic CsA nephrotoxicity presents clinically as glomerulosclerosis, tubulointerstitial fibrosis (TIF), inflammatory cell influx and tubular atrophy [14,15]. TIF, occurring as a result of chronic CsA nephrotoxicity, is irreversible and is characterised by excessive extracellular matrix (ECM) accumulation within the interstitial space, which inevitably progresses to organ failure. 

FK506 is a potent immunosuppressive agent, up to 100 times more potent than CsA, which is effective in the prevention of allograft rejection in renal transplant recipients [16,17]. The mechanism of action of FK506 is similar to that of CsA. The process is initiated by binding of the FK506 molecule to the cytoplasmic immunophilin, FK506-binding protein 12 (FKBP12). The FK506-FKBP12 complex proceeds to inhibit calcineurin phosphatase activity and the subsequent production of IL-2 and other cytokine production. Following calcineurin inhibition, both calcium-dependent signal transduction and NFAT activation are impeded, thus suppressing the transcription of several cytokines [11,18,19].

## 3. Chronic Allograft Injury (CAI)

CAI, also referred to as Chronic Allograft Nephropathy (CAN), leads to chronic allograft dysfunction manifesting clinically as a decline in renal function and the development of hypertension and proteinuria [20]. The half-life of a cadaveric renal transplant is 12–14 years, with longer survival for living-donor grafts. Progressive deterioration of function with fibrotic changes accounts for about 35%–40% of all late allograft loss [20]. The functional and structural changes observed during CAI share striking similarities with those observed in other forms of chronic progressive kidney disease. Ultimately, the progressive decrease in the functional nephron mass is the major pathophysiological process. While multiple mechanisms contribute to the reduction in nephron mass, renal tubulointerstitial fibrosis (TIF) is considered the final common mechanism leading to ESRD regardless of the initiating insult [21,22]. It has been demonstrated in both experimental and clinical studies that TIF correlates more consistently with renal functional impairment than glomerular damage [23]. TIF is characterised by the gradual loss of the tubular epithelium, progressive accumulation of fibroblasts and alpha smooth muscle actin (α-SMA)-positive myofibroblasts and accumulation of extracellular matrix (ECM) components in the tubular interstitium [24]. This accumulation occurs because of a pathological imbalance in the production and degradation of ECM. The exact mechanism by which TIF results in renal functional decline is not fully clear; however a number of factors are thought to contribute including obliteration of postglomerular capillaries, formation of atubular glomeruli, and tubular atrophy [25,26,27]. Concomitant with the development of interstitial fibrosis is the fate of the cells of the tubulointerstitium. As fibrosis progresses, tubular cells and peritubular capillaries decrease in number and ultimately disappear. Interstitial fibroblasts become activated and increase in number, and there is a notable infiltration of inflammatory cells into the interstitial compartment [28,29].

A search of the available literature demonstrates a wide variation in the terminology used to describe the process of renal allograft nephropathy. CAI was previously referred to as Chronic Allograft Nephropathy (CAN) and the disease is classified according to the Banff scale which characterises five categories of renal allograft pathology. The new classification of renal allograft rejection has limitations; hence the use of CAN has persisted in leading academic journals. Histologically, CAI is defined by “interstitial fibrosis and tubular atrophy” (IF/TA) [2,30]. Therefore *in vivo* experiments usually focus of IF/TA development as a representative model of CAI. The application of renal cell culture or *in vitro* models in the research of CAI is complicated and most studies concentrate on the analysis of immunosuppressant-induced nephrotoxicity, since the renal injury resulting from immunosuppressive regimes is often central to allograft rejection. 

## 4. Proteomics

The evolution of proteomics is a testament to the rapid progression and multidisciplinary nature of modern science. “Proteomics” is a term coined in the 1990s to describe “proteins expressed by the genome” [31] and is defined as “the systematic analysis of proteins for their identity, quantity and function” [32]. While traditional proteomic approaches involved analysis of only one protein at a time, recent developments in the field facilitate the comparison of the expression of multiple proteins simultaneously. Proteomics has expanded in the post-genomic era to incorporate many areas of research including the determination of protein function, characterisation of post-translational modifications, structural analysis, analysis of the regulation of protein activity, analysis of protein interactions and complex formation, analysis of protein trafficking and sequestration in sub-cellular compartments, protein expression analysis, analysis of signalling and metabolic pathways, drug mode-of-action and toxicity studies [33]. The study of proteins has several principal advantages over nucleic acid studies for the investigation of mechanisms in different tissues and analysis of body fluids. Firstly, it is proteins, not nucleic acids, which mediate most of the physiological functions within the cell. Secondly, proteins are regulated in complex ways, many of which do not involve changes in mRNA levels. Protein abundance can change as a result of altered transcription or mRNA stability, but also as a result of direct regulation of translation. Additional regulatory mechanisms do not depend on changes in protein abundance but rather on modification of proteins by proteolytic processing, post-translational modifications, regulated trafficking or protein interaction [34]. Therefore most forms of cellular regulation would be undetectable by cDNA or oligonucleotide array, lending strength to the use of proteomics. While the proteome is markedly more complex than the genome with regard to the number of proteins documented exceeding the number of genes documented by 10 to 20 times, the study of the proteome is a valuable tool in researching many diseases [35]. 

**Figure 1:**
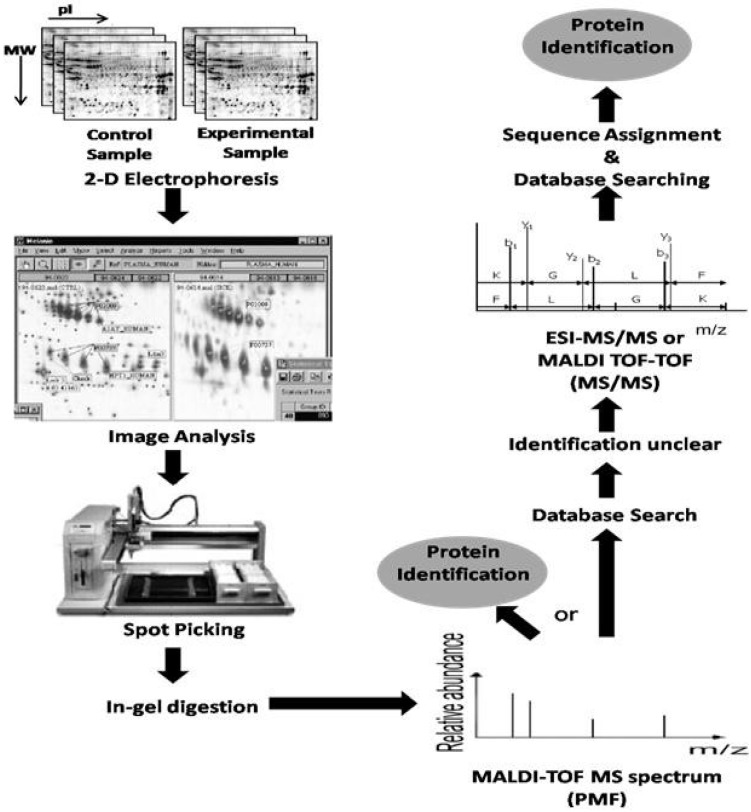
Differential display via 2-DE. A classical workflow. The samples are resolved via 2-DE in replicate gels and visualised by colloidal blue staining. Image analysis is performed using commercial software and differentially expressed protein spots are proteolytically digested from the gel for further analysis by matrix-assisted laser desorption/ionisation TOF-MS (MALDI-TOF-MS). The peptide mass fingerprint is matched against genomic or protein databases to obtain candidate proteins. Further analysis by tandem mass spectrometry (MS/MS) and peptide sequencing by analysis of fragmentation spectra aids the identification of peptides (adapted with permission from [37]).

Currently, proteomic researchers have various techniques at their disposal to facilitate human proteome studies, including gel and non-gel based approaches. Gel-based approaches typically entail a separation step, typically via 2D electrophoresis (isoelectric focusing (IEF) followed by separation based on molecular mass, followed by protein visualisation and image analysis (Figure 1). Different protein visualisation methods are available including radiolabelling, fluorescent labelling, silver staining or most commonly employed method is colloidal blue coomassie blue staining, Image analysis is constantly evolving as new computer programs are developed and improved, with some of the most popular software including Progenesis (Nonlinear Dynamics, Durham, NC, USA), Image Master 2D Platinum and Melanie Software (Amersham Biosciences, Buckinghamshire, UK), however despite recent advances mage analysis remains time-consuming. 2-DE efficiently separates complex protein mixtures producing 2D comparative protein maps which can be utilised to identify proteins expressed within a particular cell or proteins expressed as a result of a particular disease state. However, these comparative protein maps alone cannot aid in the identification of the proteins; therefore researchers combine 2D electrophoresis with mass spectrometry, such as matrix-assisted laser desorption/ionisation TOF-MS (MALDI-TOF MS), in order to identify and quantify the proteins [36]. Limitations of the gel-based approaches include low detection, sensitivity and linearity, poor solubility of membrane proteins, limited loading capacity of the gradient pH strips and low linear range of visualisation procedures [37]. 

Based on the current limitations associated with gel-based proteomic approaches, other methods have demonstrated a surge in popularity. These non-gel approaches digest the complex mixture of proteins in solution and the resulting peptide mixture is fractionated by one or several steps of capillary chromatography or HPLC prior to analysis in a data-dependant manner by MS/MS. These types of non-gel based approaches are employed in peptidomics, which refers to the study of peptides under 10 kDa. Bioinformatic approaches are central to proteomic studies, facilitating the management, data elaboration and integration of huge amounts of data. Bioinformatics is fundamental in order to reduce the analysis time and to provide statistically significant results. In order to process the data effectively, new software programs and algorithms are continuously been developed to improve protein identification, characterisation and quantification in terms of high-throughput and statistical accuracy. The most utilised non-gel proteomic approaches in the literature are LC/MS and SELDI-TOF MS. 

LC/MS, a quantitative comparison of ions emanating from identically prepared control and experimental samples, was developed using a reproducible chromatographic separation system coupled with a high mass resolution and mass accuracy of an orthogonal time-of-flight (TOF) mass spectrometer [38]. The instrument alternates between low and high collision energies in MS analysis. The low collision energy scan results in the determination of accurate precursor ion masses, while the high energy scan generates accurate peptide fragmentation data. This multiplex parallel fragmentation yields uniformly product ion information of all peptides across their entire chromatographic peaks, providing continuous MS data throughout the entire acquisition. LC/MS approach is suitable for relative and absolute quantification [39]. 

**Figure 2:**
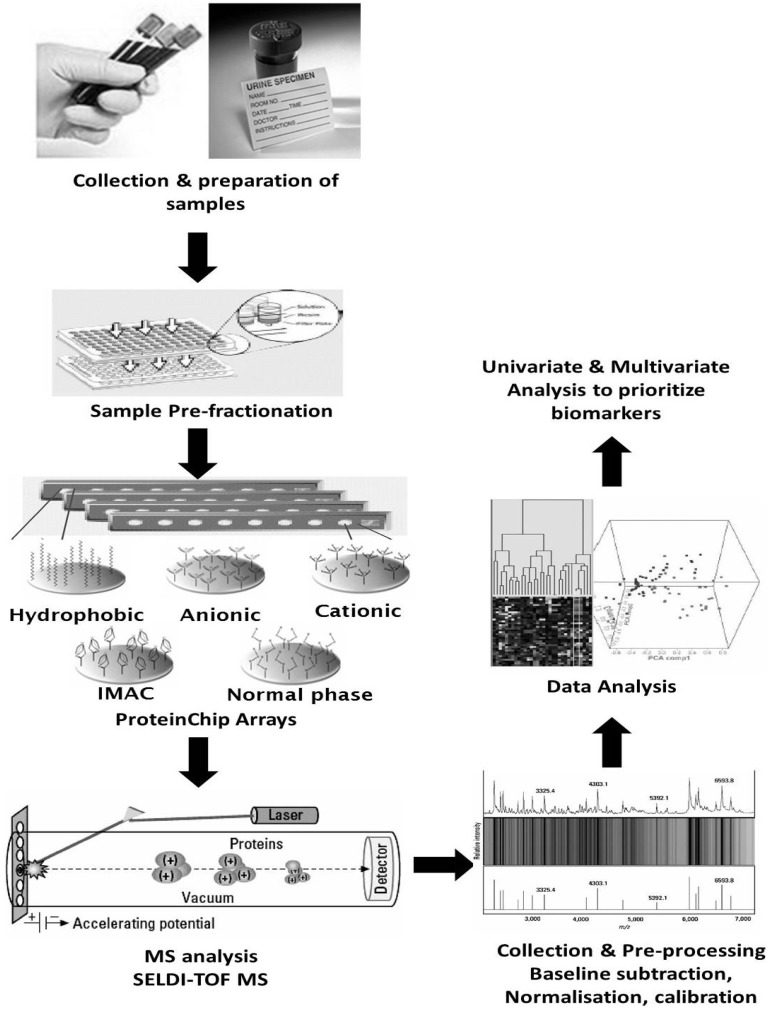
Differential protein expression profiling by Surface-Enhanced Laser Desorption/Ionization (SELDI)-TOF MS. The complex mixture was pre-fractionated chromatographically using protein chip arrays. The chips consist of 8 or 16 spots of a specific chromatographic surface (hydrophobic, cation exchange, anion exchange, metal affinity). MS spectra of bound proteins are obtained by SELDI-TOF MS. Output data from MS is displayed as trace, gel and map views. Univariate and/or multivariate analysis is performed using the appropriate software to determine differentially expressed proteins (adapted with permission from [37]).

SELDI-TOF MS facilitates analysis of complex protein mixtures separated by on-chip retentate chromatography. The sample is pre-fractionated or laded directly onto several chemically treated supports known as protein chip arrays, each with a specific chromatographic feature (anionic or cationic, hydrophilic or hydrophobic, or ion metal chelating). Protein microarray chips are designed to retain proteins based on specific physiochemical properties, aiding on-surface chromatographic protein separation.MS spectra acquisition is then conducted by co-crystallising the immobilised proteins with a matrix on a target surface and MS spectra are acquired using a specific mass analyser e.g., a SELDI-TOF mass spectrometer which generates low-resolution protein patterns of the proteins bound to the chromatographic surface (Figure 2). 

## 5. Clinical Proteomic Studies

To date several proteomic studies of CAI have been performed, the results showing promising proteomic profiles that can discriminate control patients from CAI patients, thus providing novel biomarkers of CAI for further testing. These studies have been carried out in kidney biopsies, serum and urine samples. 

### 5.1. Clinical Proteomics Using Tissue Biopsies for Biomarker Discovery

Nakorchevsky *et al.* conducted a large-scale proteo-genomic analysis of kidney transplant biopsies from patients with IF/TA [40]. Tandem mass spectrometry identified more than 1,400 proteins with unique expression profiles in biopsies with mild to severe CAI compared with normal kidney biopsies. Kidney biopsies were classified as one of four stages of IF/TA [Banff 0 (no evidence of CAI) to Banff 3 (severe CAI)]. Differentially expressed and unique proteins were assigned to either biologic function or canonical pathways and hierarchically clustered. Expression profiles for healthy biopsies were enriched for healthy biological functions including amino acid metabolism and lipid metabolism, whereas biopsies with moderate/severe CAI were enriched for pathophysiological functions such as cancer, gastrointestinal disease and metabolic disease. Enriched proteins expressed in biopsies with evidence of IF/TA are known to be involved in renal apoptosis, necrosis and renal fibrosis [40]. 

### 5.2. Clinical Proteomics Using Blood for Biomarker Discovery

A similar proteo-genomic study by Kurian and colleagues utilising tandem mass spectrometry was performed in peripheral blood lymphocytes isolated from kidney transplant patients, identifying proteomic markers of mild and moderate/severe CAI. In this particular study, 302 proteins were identified that were unique to mild CAI and these proteins were mapped to functional pathways such as calcium signalling. There were 509 proteins identified which were unique to moderate/severe CAI and which were mapped to pathways including apoptosis and natural killer (NK) cell signalling. Gene profiling identified 1,066 genes that were differentially expressed in mild CAI and 62 genes differentially expressed in moderate/severe CAI. Several hundred mRNA and proteomic biomarkers defining unique proteo-genomic signatures of CAI, with 80% class prediction accuracy for mild CAI and 92% for moderate/severe CAI [41]. 

Proteomic studies have also been used to analyse effects of drugs on peptide profiles and thus to gain insight into the mechanism of action. Perez *et al*. studied the effect of statins, administered to manage dislipidemia, on peptide profiles in renal transplant recipients. Dislipidemia occurs in more than 80% of renal transplant recipients, with immunosuppressant regimes playing a central role in disease progression [42]. In this particular study, urine samples from 29 kidney transplant patients were analysed using MALDI-TOF MS to assess whether treatment with the statin, atorvastatin, caused any modification in the urinary proteomic profile. The analysis yielded no significant differences in the urinary peptidome, although some peptides increased or decreased after treatment [43]. A later study conducted by the same group utilised MALDI-TOF MS to analyse serum samples from 54 kidney transplant patients. Results demonstrated that atorvastatin therapy caused the reduction of 11 peptide signals and an increase of 3 peptide signals. Two of the peptides that were reduced after treatment were identified as bradykinin and complement factor C4; both proteins have been shown to be involved in the inflammatory process. The results suggest that proteomic analysis could be used as a tool in investigating molecular modifications induced by drug therapy [44]. 

### 5.3. Clinical Proteomics Using Urine for Biomarker Discovery

To date the majority of proteomic studies concerned with CAI have utilised urine as the most common biological sample. Urine is the biological sample of choice for proteomic analysis in kidney disease, with normal urinary proteins generally reflecting normal tubular physiology. One of the kidneys’ main functions is the elimination of waste products, especially urea and metabolites, from the plasma. The kidneys filter large volumes of plasma (350–400 mL/100 g of tissue per minute) daily, selectively reabsorbing to a volume of less than 1% of the original ultrafiltrate which is then excreted as urine. Approximately 30% of urinary proteins are plasma proteins, whereas the other 70% are produced in the kidney [45,46]. Urinary proteomics can prove to be particularly challenging for numerous reasons. Normally the urinary protein concentration is very low; in a healthy individual the protein content less than 150 mg per day (or approximately 0.1 mg of protein/mL urine). Furthermore, a large percentage of the urinary protein content consists of serum albumin (~10%) and uromodulin or Tamms-Horsfall glycoprotein (THP) (~50%) [47], which poses a significant obstacle to MS analysis, interfering with the detection of other less abundant proteins. Urinary protein profiles display wide intra- and inter-patient variability [48]. This high degree of variability poses a challenge for the creation of a valid and reliable urinary proteome map.

Bertoni *et al.* assessed the effects of the anti-mTOR immunosuppressive drug, everolimus (EVL) on renal function post-transplantation. A proteomic approach was employed in order to characterize proteinuria in renal transplant patients who were receiving EVL. Proteinuria was evident during the first two days of treatment with EVL. Quantitative and qualitative proteomics was performed on urine samples using 2D electrophoresis and MALDI-TOF MS technologies, identifying an increase in β2-microglobulin and α1-microglobulin after treatment with EVL. Changes in proteins involved in glomerular damage were identified including albumin, Zn-α1 glycoprotein, α2HS glycoprotein and leucine-rich α2 glycoprotein. Clusters of α1-antitrypsin fragments and monoclonal λ chains were identified as specific biomarkers for EVL [49]. 

A study by O’Riordan *et al*. [50] performed urinary proteomic analysis on 75 renal transplant recipients and 20 healthy volunteers using SELDI-TOF mass spectrometry. Patients could be classified into subgroups with Banff 0 (IF/TA grade 0) and Banff 2–3 (advanced IF/TA) with a sensitivity of 86% and a specificity of 92%. Urinary proteins identified that were associated with advanced CAI including α1-microglobulin, β2-microglobulin, prealbumin and a *C*-terminal perlecan fragment, endorepellin. Further investigations into perlecan and endorepellin by means of ELISA showed increased urinary expression of endorepellin and immunofluorescence analysis of renal biopsies demonstrated increased expression of the endorepellin/perlecan ratio in renal biopsies of patients with advanced CAI [50].

A urinary proteomic study was conducted utilising MALDI-TOF to analyse the differences in the urinary patterns of 32 patients with chronic allograft dysfunction (CAD) [51]. CAD is defined as progressive graft failure with slowly rising serum creatinine and decreasing GFR. The cohort contained patients with pure IF/TA and chronic active antibody-mediated rejection (CAAR), which controls including stable graft recipients and healthy control subjects. Unsupervised hierarchical clustering highlighted good segregation of samples into the four distinct biomedical groups. Additionally, the proteome of the pure IF/TA group varied from that of the CAAR group, a result that was confirmed using an independent validation set. A pattern of 14 proteins was identified which provided the best discrimination between these two groups, correctly identifying 100% of the patients with pure IF/TA and 100% of the patients with CAAR [51]. 

A similar study performed by the same group employed a label-free quantitative LC-MS/MS strategy to quantify polypeptide ions in urine samples across 39 CAD patients, including patients with IF/TA and CAAR, and 32 control individuals. Peptides that best discriminated the two groups were derived from uromodulin and kininogen which were found to be more abundant in control than in CAD patients. As well as these two peptides, ions at *m/z* 645.59 and *m/z* 642.61 were shown to be more abundant in CAAR patients than in IF/TA and stable renal transplant, allowing discrimination between the two forms of CAD. Overall, it was confirmed the best discriminatory markers were the combination of low expression of uromodulin coupled with high *m/z* 642.61 expression which were characteristic of CAD urine [52]. 

Banon-Maneus *et al.* employed two-dimensional gel electrophoresis and MALDI-TOF-MS and capillary liquid chromatography-electrospray tandem mass spectrometry using an LTQ linear ion trap (LC-ESI-ITMS/MS) in the identification of new candidate biomarkers of CAD. The study cohort included 36 transplant recipients including individuals with stable renal transplant (IF/TA-0 group), individuals with IF/TA grade 1 and individuals with IF/TA-2-3 at least six months after transplantation. The study identified 19 proteins with differential abundance depending on the IF/TA score. Of these, 11 proteins were identified in advanced IF/TA and included β2 microglobulin, MASP-2, α-1-β-glycoprotein, leucine-rich α-2-glycoprotein-1, α-1-antitrypsin, gelsolin precursor, apoptosis-inducing factor-like mitochondrion-associated inducer of death, heparan sulphate proteoglycan, anti-TNF-α antibody light-chain and dimethyl-arginine dimethylaminohydrolase-2 [53].

A study conducted in 2012 by Tetaz *et al.* on urine samples obtained from 29 patients three months post-transplantation employed SELDI-TOF MS to identify predictive urinary biomarkers of CAD. CAD development was confirmed by serum creatinine measurement and allograft biopsy one year post-transplantation. The study identified 18 biomarkers which were predictive of CAD occurrence. The biomarker with the highest diagnostic performance was a protein of 8,860Da that predicted CAD with a sensitivity of 93% and a specificity of 65%. The relevance of the individual biomarkers and a biomarker pattern consisting of three proteins was confirmed in an independent cohort of patients with undetermined CAD status one year post-transplantation [54]. 

In another study, Srivastava *et al.* used an alternative methodology to identify several candidate biomarkers that discriminate between patients with acute and chronic allograft rejections. Using large-scale antibody microarrays, urine samples from a cohort of 37 patients (5 normal controls, 11 patients with stable function, 11 patients with chronic graft injury and 10 patients with acute rejection); 12 proteins were identified which were elevated in the two rejection stages. Reverse phase protein microarrays were performed to qualify some of these signals and candidates were identified which either individually or collectively contributed to a urine proteomic signature for diagnosis of allograft rejection, these candidates included ANXA11, Integrin β3, Integrin α3 and TNFα [55]. 

**Figure 3:**
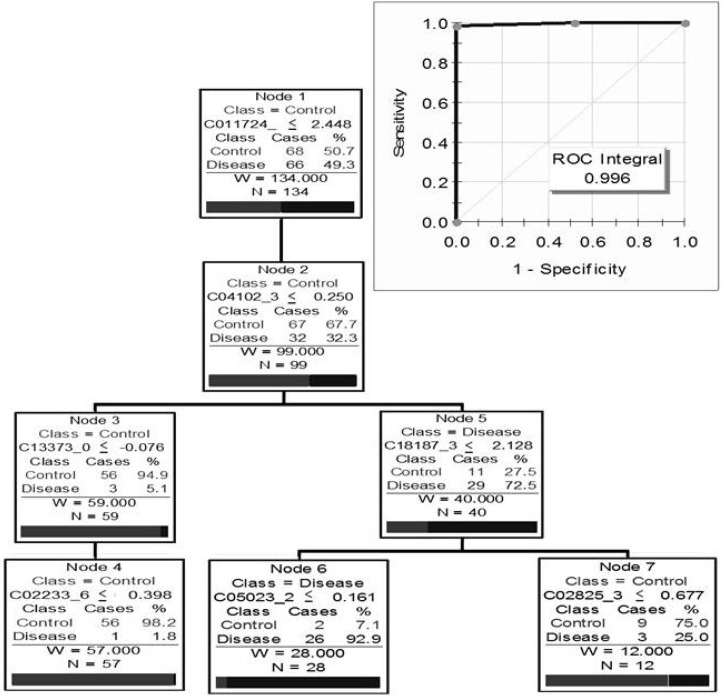
Optimal biomarker pattern tree for detecting chronic allograft nephropathy (CAN) in transplant patient urine and Receiver Operator Characteristic curve for this tree using a **CM10** ProteinChip^TM^ (adapted with permission from [56]).

**Figure 4:**
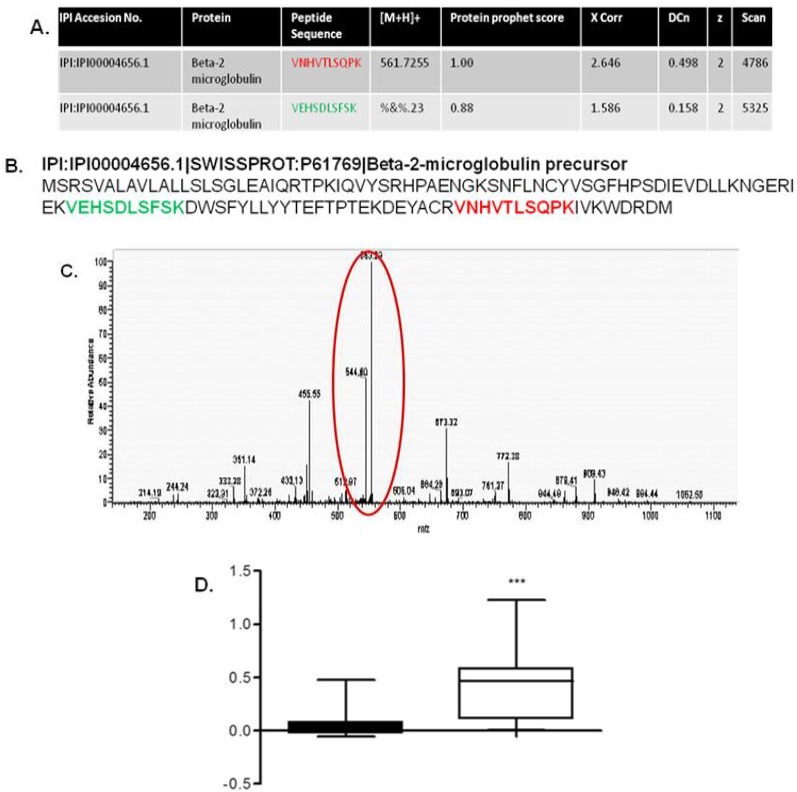
Identification of β2 microglobulin. (**A**) Two high-scoring tryptic peptides; (**B**) were matched to the sequence of β2 microglobulin; (**C**) A representative spectrum of the [M+2H]^2+^ ion from peptide VNHVTLSQPK is shown; (**D**) Urine β2 microglobulin quantification by ELISA in chronic allograft nephropathy patients (adapted with permission from [56]).

Finally, a study conducted in our own lab by Johnston *et al.* utilised a proteomic approach to investigate a cohort of renal transplant patients for potential biomarkers specific to CAN [56]. The clinical cohort was comprised of 70 renal transplant patients (34 renal transplant patients with histologically proven CAN and 36 renal transplant patients with normal renal function). Analysis using SELDI-TOF MS identified a pattern of seven proteins with the capability to distinguish between CAN and renal transplant patients with good renal function (Figure 3). Subsequently the primary protein of this biomarker pattern was identified as β2-microglobulin (Figure 4), with significantly higher levels of this protein found in the urine of patients with CAN compared to urine of normal renal function transplant recipients (Figure 4d) [56]. 

## 6. *In Vivo* Proteomic Studies

Animal models have been used in the study of disease for many decades [57], employing a wide range of animals from rodents to primates [58,59]. Animal models offer numerous advantages over traditional cell culture systems; systemic effects can be observed, the cells have not been transformed artificially, and the replenishing of food and removal of waste products occurs continuously. One of the main disadvantages associated with animal models arises from species variability [60]. Thus, animal models have proven to be quite useful and have yielded interesting results that may have the possibility of transferring over to the clinical setting.

A study conducted by Reuter and colleagues utilised the Dark Agouti (DA) rat model as a model of IF/TA [61]. DA kidneys were allogenically transplanted to Wistar Furth (DA-WF, aTX) rats. DA kidneys transplanted to another DA rat served as controls (DA-DA-sTX). Proteomic analysis by 2D electrophoresis analysed and compared protein expression patterns of sTX and aTX kidney homogenates. In contrast to sTX, the control (aTX) rats developed IF/TA-dependent damage. 2D gel electrophoresis and mass spectrometry identified ten differentially regulated proteins; five proteins were related to oxidative stress (aldo-keto reductase, peroxiredoxin-1, NAD^+^-dependent isocitrate dehydrogenase, iron-responsive element-binding protein-1, and serum albumin). Two of the identified proteins are involved in cytoskeleton organisation (L-plastin and ezrin) while the remaining three are linked to metabolic functions (creatine kinase, ornithine aminotransferase, and fructose-1,6-bisphosphotase). It was suggested that these proteins may represent novel therapeutic targets for IF/TA [61]. 

Klawitter *et al.* utilised a Wistar rat model to investigate the effects of an array of immunosuppressive agents (CsA, FK506 and sirolimus) on renal function over a period of 28 days [62]. Proteomic analysis of whole kidney homogenates by means of 2D gel electrophoresis and subsequent analysis of the tryptic digests by LC-MS/MS showed substantial changes in protein expression following immunosupressant exposure. 75 significant protein spot changes were identified, equating to 37 individual proteins. Seven of the spots identified represented the major urinary protein α2-microglobulin. Other proteins identified included cytoskeletal proteins (such as vimentin, caldesmon and actin binding protein 1 (ABP1), actin related protein 3 (ARP3) and plastin 3T isoform); proteins involved in protein homeostasis (including regucalcin and calbindin); proteins involved in mitochondrial dysfunction (NADH-ubiquinone and oxidoreductase); proteins involved in cell metabolism (AGAT, kidney aminoacylase (KA), pyruvate kinase and fructose-1,6-biophosphotase). Several potential protein biomarkers for the glomerular and tubular damage caused by the CNIs alone and in combination were identified in this study but further analysis would be required to establish the suitability of these potential markers in kidney tissues or transplant patients [62].

**Figure 5:**
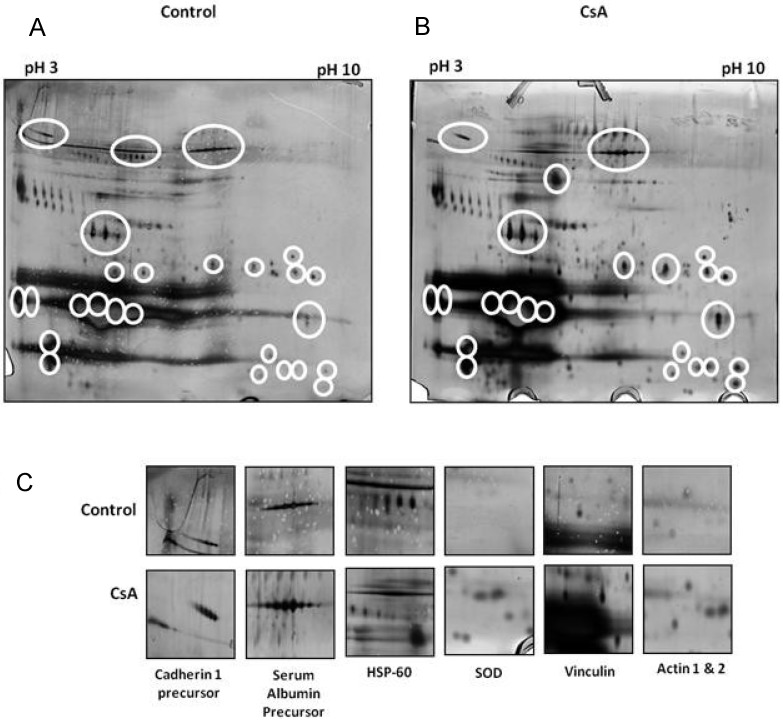
Analysis of urinary proteins in a cyclosporine A (CsA)-induced mouse model of calcineurin inhibitor (CNI) nephrotoxicity by 2D gel electrophoresis. (**A**) CsA exposure for one week, and (**B**) CsA exposure for four weeks; (**C**) Individual proteins identified from 2D screening. White circles indicate areas of differential expression (adapted with permission from [63]).

Another such study [63] involved the study of CsA administration in the CD-1 mouse model and proteomic analysis over a 4 week period. The study utilised 2D electrophoresis and mass spectrometry and through these methods 15 urinary proteins were found to be altered after week 1 of the study. These included heat shock protein 60 (Hsp60), superoxide dismutase (SOD), apolipoprotein A1, and serum albumin precursor and CDH-1 (Cadherin 1) (Figure 5). CDH-1 is an epithelial adhesion molecule that has a key role in the tubular epithelium. The loss of this protein in the urine suggests that there is tubular junction damage and breakdown and thus a loss of tubular function. Interestingly another study [64] found that this may be a marker in diabetic patients and so this furthers the hypothesis that this molecule may have biomarker potential. 

## 7. *In Vitro* Proteomic Studies

The application of *in vitro* techniques has heralded major advances in the scientific understanding of normal and disease-associated renal cell function, epithelial cell biology, and morphogenesis. In-depth studies of the properties of glomerular epithelial cells, endothelia and mesangial cells, as well as the numerous other epithelial cell types located within the proximal, distal and collecting tubules, are essential in establishing a fundamental understanding of normal kidney function and development, as well as offering insights into mechanisms of injury and disease [65,66]. Researchers today are presented with a wide choice of *in vitro* models, such as the isolated perfused kidney [67], tissue slices [68,69], isolated renal tubules [70] and glomeruli [71]. One of the main advantages of the aforementioned models is that the cells retain their original architecture and display their major characteristics as evidenced *in vivo*. Conversely, these models are limited in their scope as the preparations are short lived and any response observed cannot be attributed to a specific cell type. To combat these shortcomings individual cell preparations, in the form of either primary cells or established cell lines, have been developed using tissue culture techniques. These types of cell models facilitate the study of various responses in one particular cell type in situations reminiscent of both normal and disease conditions [65]. *In vitro* renal cell culture offers a valuable platform for the assessment of toxicity and investigations into the mechanisms involved in nephrotoxicity [72]. It also allows the analysis of potential markers of disease or toxicity development and the mechanisms involved, which may lead to the identification of novel therapeutic targets and biomarkers. *In vitro* models have also provided some insight into potential early stage biomarkers of CAI which could play important roles in future biomarker discovery. 

Lamoureux *et al*. conducted an *in vitro* study of CsA *vs*. FK506-induced toxicity using the human embryonic kidney cell line, HEK-293 [73]. Stable isotope labelling with amino acids in cell culture (SILAC) was used to investigate which proteins were quantitatively modified in a renal cell line exposed to clinically relevant CsA or FK506 concentrations. HEK-293 cells were cultured either in complete “heavy” or “light” DMEM media prior to immunosuppressant exposure for 24 h. Proteins were extracted and trypsin digested and purified by SPE prior to being separated by nano-scale liquid chromatography using an Ultimate 3000 HPLC system connected with an online plate spotting system. Proteins were identified using MALDI-TOF mass spectrometry. More than 650 proteins were identified in cell lysates, of which 494 proteins were identified in at least two SILAC experiments. Of these identified proteins, 70 proteins were shown to be significantly altered following CsA treatment. Upregulated proteins are known to be involved in various stress responses, e.g., endoplasmic reticulum (ER) stress, redox homeostasis or apoptosis; regulation of the actin cytoskeleton; and metabolism/transport processes. The down-regulated proteins include several nuclear and RNA processing proteins, metabolism enzymes and cellular components. The toxicity profile varied greatly upon treatment with CsA or FK506, but similar to CsA, FK506 resulted in increased concentration of the pro-apoptotic protein Cathepsin D. FK506 did not induce overexpression of ER chaperones including ER stress markers; nor did FK506 treatment induce the down-regulation of various nuclear proteins observed in CsA treatment. This highlights a differential effect between the two drugs. This differential proteomic analysis revealed that the extracellular levels of peptidyl-prolyl cis-trans isomerase A (PPIA) and particularly peptidyl-prolyl *cis*-*trans* isomerase B (PPIB) were substantially increased following CsA exposure. Since this effect was not detected following FK506 treatment it can be deduced that these enzymes may beinvolved in CsA-related nephrotoxicity. This finding may provide new and important insights into CsA-related toxicity observed in CAI; these enzymes could represent potential new biomarkers for monitoring transplant recipients [73].

Another *in vitro* study conducted in HEK-293 cells, used a proteomic approach to uncover the cellular protein response to exposure to a clinical dose of mycophenolic acid (MPA) [74]. 2DE gels were used to separate total cell lysates following treatment with DMSO and MPA. The differentially expressed proteins were in-gel trypsinised and identified by QTOF-MS/MS analysis. Several proteins were identified with modified expression in response to MPA treatment which could potentially broaden our understanding of the adverse effects of MPA treatment in transplant recipients. Statistical analysis showed that a total of twelve proteins were shown to exhibit significantly altered expression following MPA treatment. The up-regulated spots under MPA treatment were identified as complement component 1Q subcomponent binding protein (C1q), electron transfer flavoprotein subunit beta, cytochrome b-c1 complex subunit, thioredoxin domain-containing protein 12, myosin regulatory light chain 2 (MLC2), peroxiredoxin1 (Prdx1) and profilin1. Five proteins demonstrated down-regulated expression and were identified as protein SET, stathmin, 40S ribosomal protein S12, histone H2B type 1-A and histone H2B type 1-C/E/F/G/I. Further investigations of the identified proteins may provide new insights into the cellular pathways influenced by MPA regimes and could help in more informed employment of MPA in transplantation medicine [74].

Puigmulé *et al*. conducted a differential proteomic analysis of CsA induced toxicity in renal proximal tubule cells, employing a human cell line (HK-2) and mouse cell lines (PCT3 and PR10) [75]. Exposure of the proximal tubular derived cell lines to increasing doses of CsA (0–50 μM) was performed to determine the CsA dose which induced changes in the proteomic profile in a sensitive manner. Robust and consistent proteomic profile alterations were first observed at the 10 μM dose. Overall, 38 proteins demonstrated differential expression in the CsA treated mouse PCT3 and human HK-2 cells relating to protein metabolism, response to damage, cell organisation and cytoskeleton, cell cycle, nucleoditic metabolism and energy metabolism. 1D and 2D western blot analysis of crude extracts derived from CsA-treated cells or kidneys with impaired function following CsA treatment revealed a correlation with proteomic changes or differential isoform expression in randomly selected proteins. Proteins highlighted in this study may prove to be useful biomarkers which could eventually identify CsA toxicity in CAN using protocol biopsies of transplanted patients, thus facilitating the adjustment of CsA doses to non-toxic ranges [75].

## 8. Discussion

This review concentrated on proteomic approaches to biomarker elucidation in long-term renal allograft outcome and the development of CAI. Proteomics-based approaches have generated an impressive quantity of data in the renal field; although at present the scope of proteomic studies in CAI-based research have been limited. New biomarkers of renal allograft rejection are required in the clinic; where the early and reliable detection of immunosuppressant-induced nephrotoxicity following kidney transplant would minimize the current rates of graft loss, vastly improving the quality of life of patients. 

Limited *in vivo* and *in vitro* studies have been conducted in this particular area, with the *in vivo* studies focussing on the development of IF/TA while the *in vitro* studies are concerned with the detection of immunosuppressant- induced nephrotoxicity, both occurrences central to the development of CAI in transplant patients. The published *in vivo* studies suggest a role for several different regulatory pathways in IF/TA development; differentially expressed proteins were linked to cytoskeleton organisation (plastins), oxidative stress (peroxiredoxin-1, superoxide dismutase), metabolic functions (fructose-1,6-biphosphatase), protein homeostasis and mitochondrial dysfunction. These alterations in established pathways may indicate potential novel therapeutic targets for IF/TA. The *in vitro* studies highlighted disruptions in similar pathways, with differentially expressed proteins related to stress responses (ER Stress, redox homeostasis and apoptosis), cytoskeleton organisation, metabolism and transport processes. The identification of similar proteins or regulatory pathways in both models indicates that the proteins may prove to have potential as biomarkers of CAI development and warrant further investigation in a clinical setting. 

Based on the physiological and functional aspects of the kidney, the majority of renal proteomic studies, particular CAI studies, are focused on urinary proteomics. As a biofluid, the urine composition is highly reflective of the overall health of the kidney, so changes in the urinary protein composition would be indicative of incidences of renal injury or renal disease onset. CAN or CAI is one area of renal research in which there are limited publications to date regarding the identification of urinary biomarkers of CAN or renal transplant dysfunction, with most studies identifying biomarker patterns indicative of CAN [53,56]. Published CAI proteomic studies to date have identified several potential biomarkers of the disease, including α1-microglobulin, β2-microglobulin, α1-antitrypsin, albumin, uromodulin and kininogen. Many of these potential biomarkers have already been established as biomarkers in other forms kidney disease, adding credence to their use in monitoring renal graft function in transplant patients. 

## 9. The Future of Proteomics in CAI Biomarker Identification

Since the advent of renal transplantation, numerous attempts have been made to identify non-invasive biomarkers of rejection and CAI development. Unfortunately, as of yet, none of the identified biomarker proteins have made their way into routine clinical practice, suggesting that the “ideal biomarker” still remains undiscovered. The practical value of a new biomarker is tied to its sensitivity and specificity, it must be capable of differentiating between patients with good renal function and those presenting with CAI. Current theories suggest that it is unlikely that one single biomarker will fulfil all these requirements. A combined set of biomarkers could better reflect the heterogeneous process of rejection. From a purely statistical point of view, combinations of markers, similar to that described by Johnston *et al.* [56], with their individual sensitivities and specificities would increase the overall sensitivity and specificity. Despite many technical challenges associated with proteomic analysis, progress is steadily being made and new markers will emerge as momentum gathers in the field of CAI. These proteomic analyses of CAI could provide potential mechanistic insights into the origins of the disease and aid the discovery of new biomarkers or biomarker patterns with the potential to indicate disease progression, diagnosis or individual patient response to treatments. Therefore the future is bright for proteomics in biomarker identification, not only in the elucidation of novel biomarkers for the detection of the onset of renal disease but also biomarkers capable of differentiating the particular disease stage.

## References

[1] Andoh T.F., Bennett W.M. (1998). Chronic cyclosporine nephrotoxicity. Curr. Opin. Nephrol. Hypertens..

[2] El-Zoghby Z.M., Stegall M.D., Lager D.J., Kremers W.K., Amer H., Gloor J.M., Cosio F.G. (2009). Identifying specific causes of kidney allograft loss. Am. J. Transplant..

[3] Ishii Y., Sawada T., Kubota K., Fuchinoue S., Teraoka S., Shimizu A. (2005). Loss of peritubular capillaries in the development of chronic allograft nephropathy. Transplant. Proc..

[4] Nankivell B.J., Chapman J.R. (2006). Chronic allograft nephropathy: Current concepts and future directions. Transplantation.

[5] Akalin E., O’Connell P.J. (2010). Genomics of chronic allograft injury. Kidney Int. Suppl..

[6] Wolfe R.A., Ashby V.B., Milford E.L., Ojo A.O., Ettenger R.E., Agodoa L.Y., Held P.J., Port F.K. (1999). Comparison of mortality in all patients on dialysis, patients on dialysis awaiting transplantation, and recipients of a first cadaveric transplant. N. Engl. J. Med..

[7] Suthanthiran M., Strom T.B. (1994). Renal transplantation. N. Engl. J. Med..

[8] Textor S.C., Wiesner R., Wilson D.J., Porayko M., Romero J.C., Burnett J.C., Gores G., Hay E., Dickson E.R., Krom R.A. (1993). Systemic and renal hemodynamic differences between FK506 and cyclosporine in liver transplant recipients. Transplantation.

[9] Li C., Lim S.W., Sun B.K., Yang C.W. (2004). Chronic cyclosporine nephrotoxicity: New insights and preventive strategies. Yonsei Med. J..

[10] Clipstone N.A., Crabtree G.R. (1992). Identification of calcineurin as a key signalling enzyme in T-lymphocyte activation. Nature.

[11] Stepkowski S.M. (2000). Molecular targets for existing and novel immunosuppressive drugs. Expert Reviews in Molecular Medicine.

[12] Remuzzi G., Perico N. (1995). Cyclosporine-induced renal dysfunction in experimental animals and humans. Kidney Int. Suppl..

[13] Shihab F.S. (1996). Cyclosporine nephropathy: Pathophysiology and clinical impact. Semin. Nephrol..

[14] Mihatsch M.J., Thiel G., Ryffel B. (1988). Histopathology of cyclosporine nephrotoxicity. Transplant. Proc..

[15] Young B.A., Burdmann E.A., Johnson R.J., Alpers C.E., Giachelli C.M., Eng E., Andoh T., Bennett W.M., Couser W.G. (1995). Cellular proliferation and macrophage influx precede interstitial fibrosis in cyclosporine nephrotoxicity. Kidney Int..

[16] Kino T., Inamura N., Sakai F., Nakahara K., Goto T., Okuhara M., Kohsaka M., Aoki H., Ochiai T. (1987). Effect of FK-506 on human mixed lymphocyte reaction *in vitro*. Transplant. Proc..

[17] Starzl T.E., Todo S., Fung J., Demetris A.J., Venkataramman R., Jain A. (1989). FK 506 for liver, kidney, and pancreas transplantation. Lancet.

[18] Cardenas M.E., Zhu D., Heitman J. (1995). Molecular mechanisms of immunosuppression by cyclosporine, FK506, and rapamycin. Curr. Opin. Nephrol. Hypertens..

[19] Timmerman L.A., Clipstone N.A., Ho S.N., Northrop J.P., Crabtree G.R. (1996). Rapid shuttling of NF-AT in discrimination of Ca^2+^ signals and immunosuppression. Nature.

[20] Sijpkens Y.W., Doxiadis I.I., van Kemenade F.J., Zwinderman A.H., de Fijter J.W., Claas F.H., Bruijn J.A., Paul L.C. (2003). Chronic rejection with or without transplant vasculopathy. Clin. Transplant..

[21] Iwano M., Neilson E.G. (2004). Mechanisms of tubulointerstitial fibrosis. Curr. Opin. Nephrol. Hypertens..

[22] Liu Y. (2010). New insights into epithelial-mesenchymal transition in kidney fibrosis. J. Am. Soc. Nephrol..

[23] Nath K.A. (1998). The tubulointerstitium in progressive renal disease. Kidney Int..

[24] Masszi A., Fan L., Rosivall L., McCulloch C.A., Rotstein O.D, Mucsi I., Kapus A. (2004). Integrity of cell-cell contacts is a critical regulator of TGF-beta 1-induced epithelial-to-myofibroblast transition: Role for beta-catenin. Am. J. Pathol..

[25] Kang D.H., Kanellis J., Hugo C., Truong L., Anderson S., Kerjaschki D., Schreiner G.F., Johnson R.J. (2002). Role of the microvascular endothelium in progressive renal disease. J. Am. Soc. Nephrol..

[26] Marcussen N. (1995). Atubular glomeruli in chronic renal disease. Curr. Top. Pathol..

[27] Strutz F., Zeisberg M., Ziyadeh F.N., Yang C.Q., Kalluri R., Muller G.A., Neilson E.G. (2002). Role of basic fibroblast growth factor-2 in epithelial-mesenchymal transformation. Kidney Int..

[28] Eddy A.A. (1996). Molecular insights into renal interstitial fibrosis. J. Am. Soc. Nephrol..

[29] Eddy A.A. (2005). Progression in chronic kidney disease. Adv. Chronic Kidney Dis..

[30] Solez K., Colvin R.B., Racusen L.C., Sis B., Halloran P.F., Birk P.E., Campbell P.M., Cascalho M., Collins A.B., Demetris A.J. (2007). Banff '05 Meeting Report: Differential diagnosis of chronic allograft injury and elimination of chronic allograft nephropathy (“CAN”). Am. J. Transplant..

[31] Wilkins M.R., Pasquali C., Appel R.D., Ou O., Golaz O., Sanchez J.C., Yan J.X., Gooley A.A., Hughes G., Humphery-Smith I. (1996). From proteins to proteomes: Large scale protein identification by two-dimensional electrophoresis and amino acid analysis. Biotechnology (NY).

[32] Peng J., Gygi S.P. (2001). Proteomics: The move to mixtures. J. Mass Spectrom..

[33] Naaby-Hansen S., Waterfield M.D., Cramer R. (2001). Proteomics—Post-genomic cartography to understand gene function. Trends Pharmacol. Sci..

[34] Knepper M.A. (2002). Proteomics and the kidney. J. Am. Soc. Nephrol..

[35] Lander E.S., Linton L.M., Birren B., Nusbaum C., Zody M.C., Baldwin J., Devon K., Dewar K., Doyle M., FitzHugh W. (2001). Initial sequencing and analysis of the human genome. Nature.

[36] Banks R.E., Dunn M.J., Hochstrasser D.F., Sanchez J.C., Blackstock W., Pappin D.J., Selby P.J. (2000). Proteomics: New perspectives, new biomedical opportunities. Lancet.

[37] Monteoliva L., Albar J.P. (2004). Differential proteomics: An overview of gel and non-gel based approaches. Brief. Funct. Genomic Proteomic.

[38] Silva J.C., Denny R., Dorschel C.A., Gorenstein M., Kass I.J., Li G.Z., McKenna T., Nold M.J., Richardson K., Young P. (2005). Quantitative proteomic analysis by accurate mass retention time pairs. Anal. Chem..

[39] Blackburn K., Mbeunkui F., Mitra S.K., Mentzel T., Goshe M.B. (2010). Improving protein and proteome coverage through data-independent multiplexed peptide fragmentation. J. Proteome Res..

[40] Nakorchevsky A., Hewel J.A., Kurian S.M., Mondala T.S., Campbell D., Head S.R., Marsh C.L., Yates J.R., Salomon D.R. (2010). Molecular mechanisms of chronic kidney transplant rejection via large-scale proteogenomic analysis of tissue biopsies. J. Am. Soc. Nephrol..

[41] Kurian S.M., Heilman R., Mondala T.S., Nakorchevsky A., Hewel J.A., Campbell D., Robison E.H., Wang L., Lin W., Gaber L. (2009). Biomarkers for early and late stage chronic allograft nephropathy by proteogenomic profiling of peripheral blood. PLoS One.

[42] Gonyea J.E., Anderson C.F. (1992). Weight change and serum lipoproteins in recipients of renal allografts. Mayo Clin. Proc..

[43] Perez V., Navarro-Munoz M., Bayes B., Lauzurica R., Pastor M.C., Troya M., Bonet J., Ibernon M., Navarro M., Serra A. (2009). Effect of low doses of atorvastatin on the urinary peptide profile of kidney transplant patients. Transplant. Proc..

[44] Perez V., Navarro-Munoz M., Mas S., Bayes B., Pastor M.C., Martinez-Caceres E., Lauzurica R., Egido J., Romero R. (2011). Proteomic approach to the study of statin pleiotropy in kidney transplant patients. Pharmacology.

[45] Thongboonkerd V., McLeish K.R., Arthur J.M., Klein J.B. (2002). Proteomic analysis of normal human urinary proteins isolated by acetone precipitation or ultracentrifugation. Kidney Int..

[46] Pieper R., Gatlin C.L., McGrath A.M., Makusky A.J., Mondal M., Seonarain M., Field E., Schatz C.R., Estock M.A., Ahmed N. (2004). Characterization of the human urinary proteome: A method for high-resolution display of urinary proteins on two-dimensional electrophoresis gels with a yield of nearly 1,400 distinct protein spots. Proteomics.

[47] Julian B.A., Suzuki H., Suzuki Y., Tomino Y., Spasovski G., Novak J. (2009). Sources of urinary proteins and their analysis by urinary proteomics for the detection of biomarkers of disease. Proteomics Clin. Appl..

[48] Kroot J.J., Hendriks J.C., Laarakkers C.M., Klaver S.M., Kemna E.H., Tjalsma H., Swinkels D.W. (2009). (Pre)analytical imprecision, between-subject variability, and daily variations in serum and urine hepcidin: Implications for clinical studies. Anal. Biochem..

[49] Bertoni E., Bruschi M., Candiano G., Boccardi C., Citti L., Mangraviti S., Rosso G., Larti A., Rosati A., Ghiggeri G.M. (2009). Posttransplant proteinuria associated with everolimus. Transplant. Proc..

[50] O’Riordan E., Orlova T.N., Mendelev N., Patschan D., Kemp R., Chander P.N., Hu R., Hao G., Gross S.S., Iozzo R.V. (2008). Urinary proteomic analysis of chronic allograft nephropathy. Proteomics Clin. Appl..

[51] Quintana L.F., Sole-Gonzalez A., Kalko S.G., Banon-Maneus E., Sole M., Diekmann F., Gutierrez-Dalmau A., Abian J., Campistol J.M. (2009). Urine proteomics to detect biomarkers for chronic allograft dysfunction. J. Am. Soc. Nephrol..

[52] Quintana L.F., Campistol J.M., Alcolea M.P., Banon-Maneus E., Sol-Gonzalez A., Cutillas P.R. (2009). Application of label-free quantitative peptidomics for the identification of urinary biomarkers of kidney chronic allograft dysfunction. Mol. Cell. Proteomics.

[53] Banon-Maneus E., Diekmann F., Carrascal M., Quintana L.F., Moya-Rull D., Bescos M., Ramirez-Bajo M.J., Rovira J., Gutierrez-Dalmau A., Sole-Gonzalez A. (2010). Two-dimensional difference gel electrophoresis urinary proteomic profile in the search of nonimmune chronic allograft dysfunction biomarkers. Transplantation.

[54] Tetaz R., Trocme C., Roustit M., Pinel N., Bayle F., Toussaint B., Zaoui P. (2012). Predictive diagnostic of chronic allograft dysfunction using urinary proteomics analysis. Ann. Transplant..

[55] Srivastava M., Eidelman O., Torosyan Y., Jozwik C., Mannon R.B., Pollard H.B. (2011). Elevated expression levels of ANXA11, integrins beta3 and alpha3, and TNF-alpha contribute to a candidate proteomic signature in urine for kidney allograft rejection. Proteomics Clin. Appl..

[56] Johnston O., Cassidy H., O’Connell S., O’Riordan A., Gallagher W., Maguire P.B., Wynne K., Cagney G., Ryan M.P., Conlon P.J. (2011). Identification of beta2-microglobulin as a urinary biomarker for chronic allograft nephropathy using proteomic methods. Proteomics Clin. Appl..

[57] Weiser R.S., Granger G.A., Brown W., Baker P., Jutila J., Holmes B. (1965). Production of acute allogeneic disease in mice. Transplantation.

[58] Pennisi E. (2007). Genetics. The geneticist’s best friend. Science.

[59] Nyachieo A., Chai D.C., Deprest J., Mwenda J.M., D’Hooghe T.M. (2007). The baboon as a research model for the study of endometrial biology, uterine receptivity and embryo implantation. Gynecol. Obstet. Invest..

[60] Whitworth J.A., Zhang Y., Mangos G., Kelly J.J. (2006). Species variability in cardiovascular research: The example of adrenocorticotrophin-induced hypertension. Clin. Exp. Pharmacol. Physiol..

[61] Reuter S., Reiermann S., Worner R., Schroter R., Edemir B., Buck F., Henning S., Peter-Katalinic J., Vollenbroker B., Amann K. (2010). IF/TA-related metabolic changes—Proteome analysis of rat renal allografts. Nephrol. Dial. Transplant..

[62] Klawitter J., Kushner E., Jonscher K., Bendrick-Peart J., Leibfritz D., Christians U., Schmitz V. (2010). Association of immunosuppressant-induced protein changes in the rat kidney with changes in urine metabolite patterns: A proteo-metabonomic study. J. Proteome Res..

[63] O’Connell S., Slattery C., Ryan M.P., McMorrow T. (2011). Identification of novel indicators of cyclosporine A nephrotoxicity in a CD-1 mouse model. Toxicol. Appl. Pharmacol..

[64] Zheng M., Lv L.L., Cao Y.H., Liu H., Ni J., Dai H.Y., Liu D., Lei X.D., Liu B.C. (2012). A pilot trial assessing urinary gene expression profiling with an mRNA array for diabetic nephropathy. PLoS One.

[65] Ardaillou R. (1996). Biology of glomerular cells in culture. Cell Biol. Toxicol..

[66] Wilson P.D. (2009). *In vitro* methods in renal research. Pediatric Nephrology.

[67] Schramek H., Willinger C.C., Gstraunthaler G., Pfaller W. (1992). Endothelin-3 modulates glomerular filtration rate in the isolated perfused rat kidney. Ren. Physiol. Biochem..

[68] Ruegg C.E., Gandolfi A.J., Nagle R.B., Krumdieck C.L., Brendel K. (1987). Preparation of positional renal slices for study of cell-specific toxicity. J. Pharmacol. Methods.

[69] Smith J.H. (1988). The use of renal cortical slices from the Fischer 344 rat as an *in vitro* model to evaluate nephrotoxicity. Fundam. Appl. Toxicol..

[70] Ruegg C.E. (1994). Preparation of precision-cut renal slices and renal proximal tubular fragments for evaluating segment-specific nephrotoxicity. J. Pharmacol. Toxicol. Methods.

[71] Potier M., L’Azou B., Cambar J. (1996). Isolated glomeruli and cultured mesangial cells as *in vitro* models to study immunosuppressive agents. Cell Biol. Toxicol..

[72] Pfaller W., Gstraunthaler G. (1998). Nephrotoxicity testing *in vitro*—What we know and what we need to know. Environ. Health Perspect..

[73] Lamoureux F., Mestre E., Essig M., Sauvage F.L., Marquet P., Gastinel L.N. (2011). Quantitative proteomic analysis of cyclosporine-induced toxicity in a human kidney cell line and comparison with tacrolimus. J. Proteomics.

[74] Qasim M., Rahman H., Oellerich M., Asif A.R. (2011). Differential proteome analysis of human embryonic kidney cell line (HEK-293) following mycophenolic acid treatment. Proteome Sci..

[75] Puigmule M., Lopez-Hellin J., Sune G., Tornavaca O., Camano S., Tejedor A., Meseguer A. (2009). Differential proteomic analysis of cyclosporine A-induced toxicity in renal proximal tubule cells. Nephrol. Dial. Transplant..

